# Antimicrobial Peptides: A Promising Solution to the Rising Threat of Antibiotic Resistance

**DOI:** 10.3390/pharmaceutics16121542

**Published:** 2024-12-02

**Authors:** Tarequl Islam, Noshin Tabassum Tamanna, Md Shahjalal Sagor, Randa Mohammed Zaki, Muhammad Fazle Rabbee, Maximilian Lackner

**Affiliations:** 1 Department of Microbiology, Noakhali Science and Technology University, Sonapur, Noakhali 3814, Bangladesh; tarequembg@gmail.com; 2 Department of Pharmacy, Noakhali Science and Technology University, Sonapur, Noakhali 3814, Bangladesh; tamannanoshin@gmail.com; 3 Department of Microbiology, Jagannath University, Dhaka 1100, Bangladesh; sagortm@gmail.com; 4 Department of Pharmaceutics, College of Pharmacy, Prince Sattam Bin Abdulaziz University, P.O. Box 173, Al-Kharj 11942, Saudi Arabia; r.abdelrahman@psau.edu.sa; 5 Department of Pharmaceutics and Industrial Pharmacy, Faculty of Pharmacy, Beni-Suef University, Beni-Suef 62514, Egypt; 6 Department of Biotechnology, Yeungnam University, Gyeongsan 38541, Gyeongbuk, Republic of Korea; 7 Department of Industrial Engineering, University of Applied Sciences Technikum Wien, Hoechstaedtplatz 6, 1200 Vienna, Austria

**Keywords:** peptide antibiotics, microorganisms, multi-drug resistance, therapeutics, peptide design

## Abstract

The demand for developing novel antimicrobial drugs has increased due to the rapid appearance and global spread of antibiotic resistance. Antimicrobial peptides (AMPs) offer distinct advantages over traditional antibiotics, such as broad-range efficacy, a delayed evolution of resistance, and the capacity to enhance human immunity. AMPs are being developed as potential medicines, and current computational and experimental tools aim to facilitate their preclinical and clinical development. Structural and functional constraints as well as a more stringent regulatory framework have impeded clinical translation of AMPs as possible therapeutic agents. Although around four thousand AMPs have been identified so far, there are some limitations of using these AMPs in clinical trials due to their safety in the host and sometimes limitations in the biosynthesis or chemical synthesis of some AMPs. Overcoming these obstacles may help to open a new era of AMPs to combat superbugs without using synthetic antibiotics. This review describes the classification, mechanisms of action and immune modulation, advantages, difficulties, and opportunities of using AMPs against multidrug-resistant pathogens and highlights the need and priorities for creating targeted development strategies that take into account the most cutting-edge tools currently available. It also describes the barriers to using these AMPs in clinical trials.

## 1. Introduction

Antibiotics have been used extensively in the food, agriculture, and other industries. They hold great promise for treating bacterial infections in humans and animals [1,2,3,4]. However, overuse and long-term usage of antibiotics might lead to antibiotic resistance, which is a serious issue throughout the world and can result in the development of superbugs [1,2]. Superbugs are strains of pathogenic bacteria, viruses, parasites, and fungi that are resistant to most of the antibiotics used to treat the infections they cause. A few examples of superbugs include methicillin-resistant *Staphylococcus aureus* (MRSA), vancomycin-resistant *S. aureus* (VRSA), multidrug-resistant *Enterococcus faecalis*, *Pseudomonas aeruginosa*, *Escherichia coli*, and so on, which are responsible for nosocomial infections and many other complicated disorders [2,3,4,5]. Multi-resistant bacteria can stem from excessive antibiotics use in humans or in animals, which has led to bans of antibiotic growth promoters, for instance, in many countries. The main problem with multi-resistant bacteria is that doctors simply run out of treatment options. Meanwhile, the emergence of drug-resistant microorganisms is posing a major threat to human health and life on a global scale [4,6]. According to the World Health Organization (WHO), diseases associated with microbial superbugs killed 700,000 people worldwide in 2019 and are expected to kill 10 million per year by 2050, making them the world’s top cause of death [7]. Therefore, finding substitutes for these conventional synthetic antibiotics is urgently needed in order to combat microbial infections causing human diseases. Some measures have already been taken, such as the ban of prophylactic antibiotics administration in many countries, but these are not sufficient to prevent the formation and spread of multi-resistant germs.

**Figure 1:**
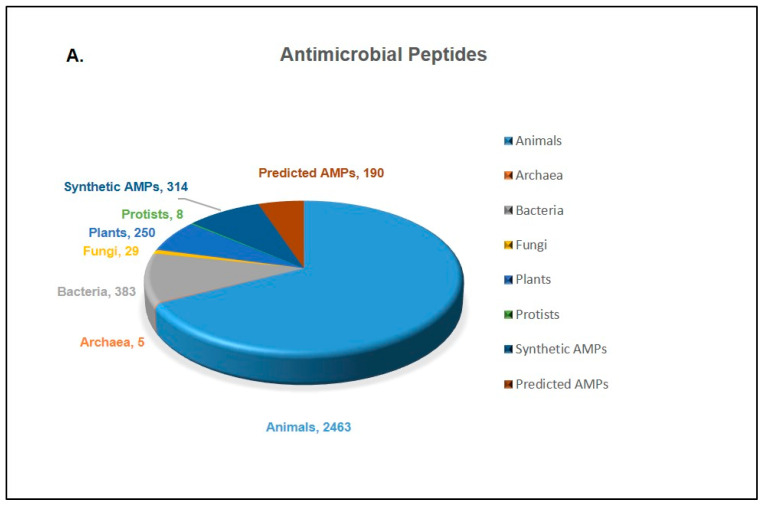

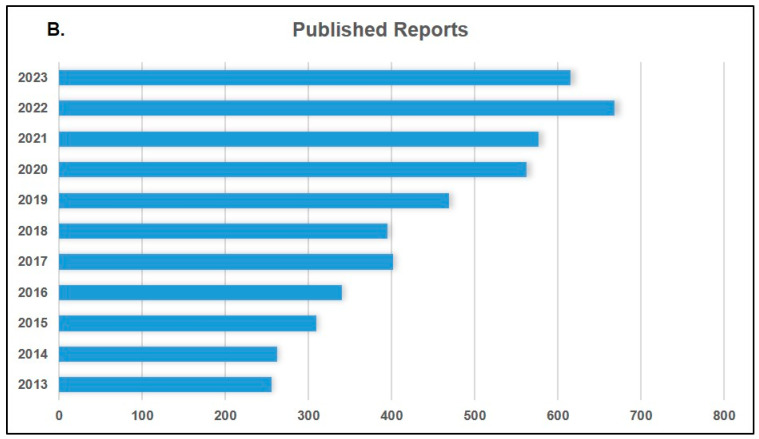
(**A**) Sources of AMPs identified by January 2024. The information was collected using APD. (**B**) Published reports on antimicrobial peptides from 2013 to 2023, including journal articles, books and documents, clinical reports, meta-analyses, and reviews. Report calculations were carried out by searching in PubMed with the help of key words “antimicrobial peptides” and “AMP”.

AMPs are tiny peptides, also called host defense peptides (HDPs), that are found in plants, animals, and microbes in large quantities. These molecules play a crucial role in the innate immune systems in all life forms. Their size may vary from 5 units to >100 units of amino acids [8]. They have broad-spectrum antimicrobial activity against a vast range of bacteria, fungi, viruses, protozoa, and even malignant cells. Large AMPs of 100 amino acids or more typically target certain microbial macromolecules, bind to nutrients, or aid in cell membrane lysis. Conversely, smaller AMPs primarily disrupt the structure and function of microbial cell membranes or directly prevent the activity of certain ATP-dependent enzymes through interactions with ATP. AMPs may also be able to improve immunity through immunomodulatory effects [9,10]. More than four thousand antimicrobial peptides (AMP) were identified in the last century according to the antimicrobial peptide database (APD: https://aps.unmc.edu/home, accessed on 8 February 2024). They belong to two major classes, ribosomal antimicrobial peptides, which are produced by all forms of life, and non-ribosomal antimicrobial peptides produced by bacteria. Antimicrobial peptides have drawn the attention of researchers for a variety of reasons. Firstly, the most interesting features lying in these AMPs are their excellent physicochemical properties; secondly, they possess a broad range of activity against microorganisms and are comparatively less susceptible to the formation of resistance [11]. As the resistance against traditional antibiotics is rising rapidly, these AMPs are great alternatives to traditional drugs [11]. The majority of the AMPs identified are from animal and plant sources, whereas the rest were identified from microorganisms (Figure 1A) [12]. Therefore, research on AMPs has increased enormously in the last decade. There has been a growing body of studies on AMPs, as seen by the research articles that have been published over the last ten years (Figure 1B). The limits of stability, toxicity, and activity associated with natural AMPs are expected to be addressed by tailored, synthetic analogues of AMPs, which have been created with certain alterations. Recombinant techniques are used to manufacture AMPs in a variety of hosts in addition to their extraction from natural sources.

Utilizing antimicrobials in combination, especially those with distinct targets, is a recognized method of combating multiple drug resistance [13]. Additionally, it permits lowering doses, lessening adverse effects, and improving chemical selectivity. The majority of AMPs have cationic properties and can adopt amphipathic conformations. This facilitates their interaction and integration of the negatively charged bacterial cell wall into the lipid bilayers [13,14]. It is also worth mentioning that a synergistic interaction with the organism’s antimicrobial peptides may really be the reason for an antimicrobial agent’s high in vivo effectiveness [15]. Conversely, finding medications that can strengthen the body’s natural defenses is interesting [16]. In certain situations, elucidating such dependencies between clinically utilized antibiotics and human AMPs may aid in enhancing the efficacy of treatment plans.

The process of developing AMPs is intricate and involves the assessment of target microorganism activity, improvement of physicochemical qualities, and picking out acceptable amino acid sequences [17]. Additionally, a major factor in the antimicrobial action of many AMPs is their amphipathic nature, which is defined by spatially separated hydrophobic and hydrophilic faces within their molecular structure [9]. Because of its amphipathicity, an AMP may be inserted into microbial membranes and yet be soluble in watery settings [18]. The short sequence length of AMPs is another important characteristic. Without sacrificing function, keeping a sequence length under 50 amino acids has benefits for more affordable chemical production, improved stability, and less immunogenicity [19]. However, designing AMPs to combat superbugs is a great challenge to researchers and scientists due to their limited stability under certain physiological circumstances, low specificity, high toxicity and hemolytic side effects, high manufacturing costs, mechanical challenges, and proteolytic breakdown [9,19]. It is possible to overcome the difficulties involved in designing AMPs for medicinal and biotechnological applications by having a better understanding of their physicochemical characteristics. The drug-like qualities and therapeutic usefulness of AMPs can be enhanced by logical optimization of these essential physicochemical features using several design strategies, including sequence changes, conformational limitations, and lipidation.

In this review, we discussed natural sources of AMPs, their classification, their mechanisms of actions based on direct killing, and immune modulation. In addition, we also discussed the role of AMPs in the host body, clinical importance, and the obstacles of using peptide antibiotics. Furthermore, we reviewed different methods for the identification or production of AMPs using different biochemical, molecular, and computer-aided methods. The focus of this review was given to the use of AMPs against pathogenic microorganisms.

## 2. Sources of Antimicrobial Peptides (AMPs)

### 2.1. Natural Sources of AMPs

Natural antimicrobial peptides (NAMPs) are members of a variety of protein or peptide families that are involved in barrier defense and innate immune responses in every aspect of life [20]. Natural remedies have been used for the management of almost 87% of all human ailments, including immune system problems, bacterial infections, and parasite infections [21,22,23]. NAMPs mostly originate from natural sources, such as bacteria, fungi, plants, protists, archaea, and mammals. Over the past 20 years, there has been an increase in interest in AMP research due to their potential activity and future prospects; as a result, the list of discovered NAMPs has been rising steadily. NAMPs are more distinctive than traditional antibiotics [20]. More than 1500 NAMPs have been found in nature, according to recent studies, and many of these NAMPs are presently undergoing preclinical or clinical trials [24]. Most of the AMPs identified are from animals and plants (Table 1), whereas the rest of them were identified from bacteria, fungi, and archaea (Table 2) [12].

The antimicrobial peptide sequence may be found using a variety of techniques, such as electro-spray ionization (ESI), nanostructure laser desorption/ionization (NALDI), and matrix-assisted laser desorption/ionization time-of-flight mass spectrometry (MALDI-TOF MS). The most popular method for identifying and characterizing peptides from diverse dietary sources is liquid chromatography combined with mass spectrometry, or LC–MS [64]. Based on the molecular mass of the analytes, LC-MS can detect and describe complex mixtures of peptide sequences with outstanding extraction efficiency and precision [65], with LC-MS/MS being an even more accurate technique [66]. Peptide databases like BIOPEP, UNIPROT, SWISS PROT, and MBPDB are used to identify amino acid sequences in order to locate novel peptides [67]. These days, in silico techniques like support vector machines, hidden Markov models, and homology modeling are being researched to verify the biological activities of identified fragments of proteins [68]. However, this traditional method of finding novel compounds is not appropriate for scale-up procedures, because it might be time-consuming, have a low peptide yield, and be expensive to produce [69]. Additionally, naturally occurring AMPs may have certain undesired properties, including poor selectivity, low hydrosolubility, hemolytic activity, host toxicity, salt sensitivity, and instability brought on by host protease degeneration [69,70].

### 2.2. Synthetic AMPs

A number of methods have been established to generate new synthetic antimicrobial peptides by changing the sequences of naturally occurring antimicrobial peptides from diverse sources. It has been shown that even slight modifications to the amino acid content can affect a peptide’s entire conformation and physicochemical makeup. The two goals of designing synthetic peptides from natural AMP sequences are as follows: (1) to extract the better portion, which has antimicrobial activity, and (2) to eliminate the worst portion, which has toxicity and low proteolysis resistance [70,71]. Usually, truncation, amino acid replacement, hybridization, and/or cyclization are used to modify the template peptide. In the large-scale synthesis of synthetic AMPs, truncating the AMP sequence to produce short peptides offers a cost-saving benefit. When AMPs are cyclized as opposed to linear peptides, the permeability of the membrane increases. Another useful tactic in the design of synthetic peptides is hybridization [72,73]. It is possible to take advantage of the various favorable qualities of template peptides by mixing snippets cut from naturally occurring AMP sequences to create hybrid peptides. For example, chimeric AMPs with strong antimicrobial activity and low toxicity can be created by combining AMPs with low toxicity and activity with AMPs that have high activity but comparatively higher toxicity [72].

Synthetic peptides are often more effective than their natural counterparts because they exhibit antibacterial activity at lower concentrations than the natural peptides from which they are derived. For instance, natural AamAP1 from *Androctonus amoreuxi* (African fattail scorpion) has antimicrobial action against *Candida albicans*, *E. coli*, and *S. aureus* at concentrations ranging from 20 to 150 μM [74]. On the other hand, synthesized forms of AamAP1, AamAP1-Lysine, are 4–20 times more potent than the natural form and have antimicrobial action against the same pathogens at lower concentrations [75].

AMPs were synthesized by using a variety of computer techniques, including genetic algorithms, machine learning techniques, language models, and motif addition techniques. These methods incorporate vital data regarding the biochemical characteristics and bioactivities of AMP sequences. As a result, before a sequence is synthesized, its antibacterial potential can be predicted. We will discuss this in more detail later in this article.

## 3. Classification of Antimicrobial Peptides (AMPs) Based on Antimicrobial Activity

**Figure 2:**
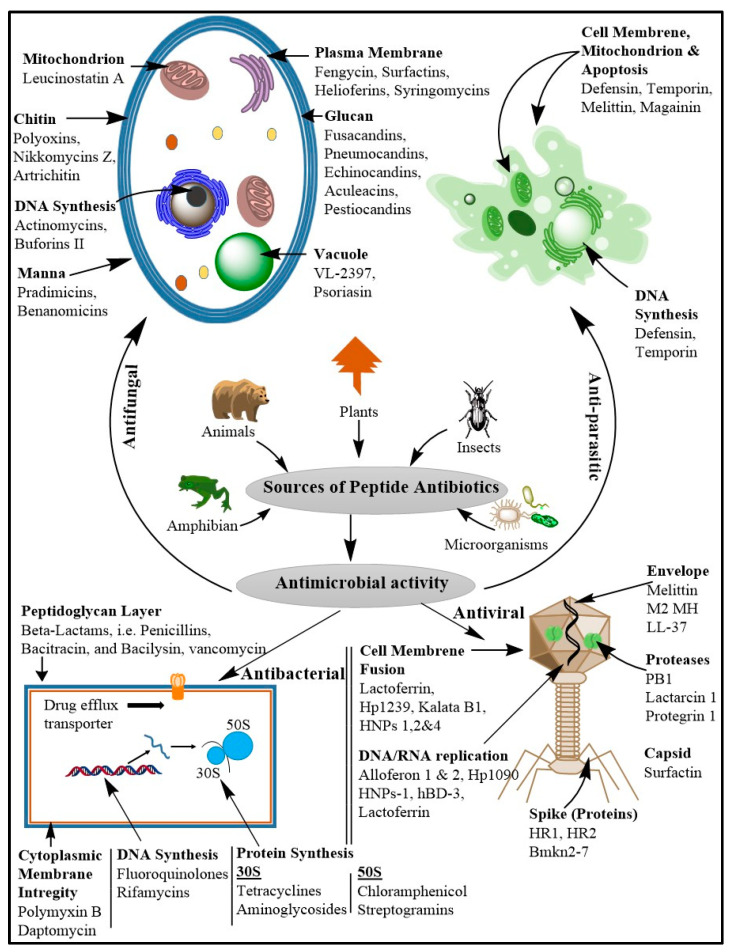
The natural sources of AMPs and their site-specific activities against different microorganisms. The figure shows different natural sources of AMPs and their antimicrobial activity against bacteria, viruses, fungi, and parasites. The bolded texts indicate the target sites of the AMPs, and the AMPs mentioned with each site indicate the AMPs that act against that specific site.

The majority of AMPs are antibacterial peptides, which account for roughly 60% of all known sequences. Next, antifungal peptides make up 26%, while the remaining 2–5% are made up of antiviral, antiparasitic, anticancer, and anti-HIV peptides [9]. Most of the antibacterial AMPs were isolated from plants, insects, and animals. In addition, microorganisms also play a vital role in producing different AMPs [12]. AMPs can be classified as based on activity, origin, structure, and amino acid-rich species. Based on the antimicrobial activity of the AMPs, these classifications can be summarized as antibacterial, antifungal, antiviral, and antiparasitic AMPs (Figure 2).

### 3.1. Antibacterial AMPs

Antibacterial AMPs are a subclass of antibiotics that are active against a wide variety of bacterial pathogens and superbugs, such as *Acinetobacter baumannii*, methicillin- and vancomycin-resistant *S*. *aureus*, *Listeria monocytogenes*, *E. coli*, *Salmonella* spp., *Vibrio parahaemolyticus*, and so on, and cause life-threatening illnesses. Most of the popular antibiotics and their derivatives that are currently used in clinical treatments, such as nisin, vancomycin, telavancin, gramicidin S, bacitracin, and polymyxin B, are produced by different bacteria and fungi [41,44,45,46]. Moreover, the AMPs P5 (YIRKIRRFFKKLKKILKK-NH2) and P9 (SYERKINRHFKTLKKNLKKK-NH2) exhibited good activity against MRSA with low cytotoxicity to human renal epithelial cells and hemocytes [76]. There are several AMPs that were discovered from bacterial or fungal origins that were not available for use until now due to the lack of safety or incomplete trials. Certain *Bacillus* spp. produce highly potent AMPs, such as bacillomycin D, fengycin, and bacilysin, which are active against a number of human pathogens as well as phytopathogens. However, these peptide antibiotics are not available for use in a formulation due to their instability outside of the host cell or in the environment, or unavailable methods to synthesize them either by chemical synthesis or by genetic modification [47,77,78].

Lycotoxin-Pa2a (Lytx-Pa2a), a novel insect AMP, was revealed from a spider venom gland transcriptome by utilizing computer-aided tactics. This AMP showed antibacterial activities by disrupting both cytoplasmic and outer membranes of pathogenic bacteria, such as Gram-negative strains (*E*. *coli* and *P*. *aeruginosa*) and Gram-positive strains (*Bacillus cereus* and *S. aureus*). In addition, this peptide antibiotic was found to induce the accumulation of reactive oxygen species and exerted no significant toxicity when brought in contact with human primary cells, murine macrophages, and bovine red blood cells [79]. In another study, RP556 showed high broad-spectrum antibacterial activity against skin and soft tissue pathogens, such as *Enterococcus faecium*, *S. aureus*, *Klebsiella pneumoniae*, *A*. *baumannii*, *P*. *aeruginosa*, and *Enterobacter* sp., without inducing cytotoxicity against Balb/c 3T3 cells, dermal fibroblasts, and hemolysis. In addition, this antibiotic initiated the production of interleukin 10 or IL-10 in LPS-induced peripheral blood mononuclear cells [80]. AMPs were also reported to inhibit bacterial infections by suppressing quorum sensing and thereby inhibiting the biosynthesis of different compounds needed for the pathogenicity of bacteria and biofilm formation. For example, octopromycin derived from octopus showed excellent antibacterial activity against multidrug-resistant *A*. *baumannii*. This molecule also aided in the suppression of biofilm formation and quorum sensing in *A*. *baumannii* [81,82]. Combinations of antimicrobial peptides were also reported to be more active against pathogens compared to single AMPs. The antibacterial activity of synthetic enterocins A, B, P, SEK4, and L50, alone and in combinations, was tested against multidrug-resistant *Clostridium perfringens*. Synthetic analogs of L50A and L50B were the AMPs with highest activities against *C. perfringens* and showed the broadest spectrum of activity against *L*. *monocytogenes*, MRSA, *Streptococcus suis*, *S*. *pyogenes*, *Enterococcus cecorum*, and *E. faecalis*, as well as Gram-negative bacteria, in particular, *Campylobacter coli* and *P*. *aeruginosa*. Synergistic combinations EntA-L50A, EntA-L50B, EntP-L50A, and EntP-L50B were found to be most active against *C. perfringens* MLG3111 [83].

### 3.2. Antifungal Peptides (AFPs)

Numerous AFPs have demonstrated outstanding antifungal properties against common pathogenic fungi, including yeast, filamentous fungi (such as *Aspergillus flavus*), and molds in food and agriculture, and *Aspergillus* and *C. albicans* in clinical medicine. In addition to brevinin, ranatuerin, and cecropins, many other synthetic peptides have potent antifungal effects. For instance, AurH1, which is derived from aurein 1.2, can be used to effectively treat *C*. *albicans* infections, which have a 40% fatality rate [84]. *A*. *flavus* produces aflatoxin, a carcinogen that is detrimental to human health. Exposure of *A*. *flavus* to multiple AFPs inhibits its growth. For instance, an AFP containing the sequence FPSHTGMSVPPP can inhibit *A. flavus* MD3 [85]. Fresh maize seeds treated with a combination containing 37 antifungal peptides extracted from *Lactobacillus plantarum* TE10 can inhibit the production of *A. flavus* spores [85]. Additionally, two chemically synthesized AMPs from radish showed strong inhibitory activity against a variety of yeast species, including *Zygosaccharomyces rouxii* and *Zygosaccharomyces bailii* [86]. *Cryptococcus neoformans* causes high levels of morbidity and mortality. Chimeric peptides made of bovine lactoferricin (LfcinB) (20–25): RRWQWR and buforin II (BFII) (32–35) have antifungal activity in vitro. RLLR sequences demonstrated strong antifungal activity against *C. neoformans* var. *grubii* with an MIC ranging from 6.25 to 12.5 µg/mL, indicating their potential as antifungal therapeutics in the future [87] (MIC = minimum inhibitory concentration). Chimeric peptides made of combining bovine lactoferricin (LfcinB) and buforin II (BFII) showed excellent antifungal activity against *Candida* spp. Chimeric peptides C9: (RRWQWR)2K-Ahx-RLLRRRLLR and C6: KKWQWK-Ahx-RLLRRLLR exhibited the strongest antifungal activity against *C. albicans* SC5314 and *C. albicans* 256, a fluconazole (FLC)-sensitive and an FLC-resistant reference strain. Chimeric peptide C9 showed MIC = 50 µg/mL (15 μM); C6 showed 50 µg/mL (24 µM) [88]. In addition to antifungal activity, these chimeric peptides also exhibited strong antibacterial activity against Gram-positive and Gram-negative bacteria, proving their efficacy as therapeutic agents for broad-spectrum antimicrobial uses [88]. Plant-derived AMPs also show excellent antifungal activity against different plant and human fungal pathogens. A cysteine-rich antimicrobial peptide (GASA/Snakin) derived from avocado fruit (*Persea americana*) showed antifungal activity against phytopathogens *Colletotrichum gloeosporioides* and *Fusarium oxysporum* and human pathogens *C. albicans* and *C. glabrata* at a concentration of 200 μg/mL [89].

### 3.3. Antiviral Peptides

Viruses seriously impair human life and cause the animal husbandry industry to suffer enormous financial losses. The latest COVID-19 epidemic has resulted in significant loss of life and property damage. In addition, there are persistent risks to human life from the foot-and-mouth disease virus, avian influenza virus (AIV), and HIV [90,91,92]. Therefore, finding solutions to these issues is crucial, and antiviral peptides provide fresh approaches. Antiviral peptides primarily kill viruses by preventing virus attachment and cell membrane fusion, damaging the virus envelope, restricting virus replication, and finally by inactivating the viral particles [93] (Figure 2). For example, AMP Epi-1 has strong antiviral activity against the foot-and-mouth disease virus and helps with the inactivation of viral particles through suppressing viral multiplication [91]. Furthermore, swine intestinal AMP (SIAMP)–IBV mixed solution inoculation significantly decreased the mortality of chicken embryos when compared to the IBV infection group, demonstrating the good inhibitory activity of SIAMP on IBV. Infectious bronchitis virus (IBV) is the pathogen of infectious bronchitis [94]. A subtype of antiviral peptides are anti-HIV peptides. Defensins (including α- and β-defensins, which have different mechanisms), LL-37, gramicidin D, caerin 1, maximin 3, magainin 2, dermaseptin-S1, dermaseptin-S4, siamycin-I, siamycin-II, and RP 71955 are among the most significant examples of these peptides [84]. The antiviral peptide enfuvirtide (Fuzeon™) has been commercialized as an anti-HIV medication [90].

Moreover, mice treated with rhesus theta-defensin 1 (RTD-1) exhibit a significant decrease in mortality when SARS-CoV is present, despite the fact that the peptide alone causes airway inflammation and that RTD-1’s sole potential mode of action is immunomodulation [95]. Intranasal delivery is the primary way of animal experimentation, and there are no clinical studies for this medication. This serves as a reminder that AMPs may benefit from nasal drug delivery (NDD) as an anti-coronavirus treatment. In addition, several antiviral peptides are included in the antiviral database AVPdb (https://webs.iiitd.edu.in/raghava/satpdb/catalogs/avpdb/, accessed on 13 February 2014).

### 3.4. Antiparasitic Peptides

The demand for innovative therapies has grown due to the rise in medication resistance against parasites. Antiparasitic peptides kill the parasites that cause diseases, like leishmaniasis and malaria [96,97]. For instance, cathelicidin and temporins-SHd have strong inhibitory action against parasites [98]. *Trichomonas vaginalis* may be effectively inhibited by a marine synthesized AMP Epi-1 by damaging its membrane [99]. Synthetic 4-amino acid peptide lysine, aspartic acid, glutamic acid, and leucine (KDEL) and the peptide Jellein, which is generated from bee royal jelly, have demonstrated an impressive effect on the *Leishmania* parasite [100,101]. Leishmanicidal peptides have been reported in a variety of organisms. For instance, halictine-2, derived from the venom of honeybees, has demonstrated significant anti-leishmanial activity without hemolytic activity for mouse macrophages and human erythrocytes [102]. Attacin, cecropin, and defensin 2 from *Lutzomyia longipalpis* (sandfly), via the Toll and Imd pathways, respond to *Leishmania infantum chagasi* infection [102]. Lastly, dragomide E is a linear lipopeptide that was obtained from the cyanobacterium *Lyngbya majuscula* and has antileishmanial activity against *Leishmania donovani* promastigotes. Furthermore, a peptide called LZ1, which is obtained from snake cathelicidin, demonstrated a potent reduction in *Plasmodium falciparum* blood stage by selectively preventing the generation of adenosine triphosphate (ATP) in erythrocytes infected with the parasite [103]. The antiparasitic activity of cyanobacterial peptides is dependent on particular protein targets, which sets them apart from AMPs from higher eukaryotes. Because of this, even if these target parasites are members of the same genus, they can be properly identified [104].

## 4. Mechanisms of Action of Antimicrobial Peptides (AMPs)

AMPs exert their antimicrobial action through two mechanisms: immune modulation and direct killing [105]. In the direct killing mechanism, there are some differences between the mechanisms of action of antimicrobial peptides with mammalian cells and microbial cells. This difference is mainly due to the difference in net charge existing on the mammal cells and microbial cells. The zwitterionic phospholipids phosphatidylethanolamine, phosphatidylcholine, and sphingomyelin are abundant in the cytoplasmic membrane of mammalian cells, giving the membrane a neutral net charge [106,107]. Hydrophobic interactions, which are comparatively weaker than electrostatic contacts between AMPs and microbial membranes, are the primary mode of interaction between AMPs and mammal cell membranes. Therefore, the AMPs are less toxic to the mammal cells. Moreover, the substantial cholesterol that makes up mammalian cell membranes—unlike that of microbes—reduces the action of AMPs by stabilizing the phospholipid bilayer [106,108,109].

The antibacterial activity of AMPs is dependent on cell membrane contact, regardless of whether translocation is necessary to reach an intracellular target or the membrane itself is the target [105,110,111]. The phospholipids phosphatidylglycerol, cardiolipin, and phosphatidylserine, which have negatively charged head groups, are strongly attracted to positively charged AMPs. This type of AMP is abundant in the cytoplasmic membranes of both Gram-positive and Gram-negative bacteria [106,107]. Additional electronegative charge is added to the bacterial surface by lipopolysaccharides (LPS) in the outer membrane of Gram-negative bacteria and teichoic acids in the cell walls of Gram-positive bacteria [107,108].

The “barrel-stave model” states that the peptides insert straight into the bilayer and that additional peptide engagement results in the formation of a peptide-lined transmembrane pore [112]. The hydrophobic side of the peptides in this pore is oriented toward the membrane’s lipid core, while the hydrophilic portions face the pore’s interior [113]. The “toroidal-pore model” states that when peptides are inserted, the phospholipid is forced to continually bend from one leaflet to the next, creating a pore that is bordered by both peptides and the phospholipid head groups [62]. The “carpet model” explains how bilayer stress, which is brought on by peptide accumulation on the membrane surface, leads to membrane rupture and the formation of micelles [105,106,114]. Membrane permeabilization is crucial for the translocation of some AMPs into the cytoplasm, where they target essential cellular functions, such as enzymatic activity, protein folding, DNA/RNA and protein synthesis, and/or cell wall synthesis, in addition to causing membrane dysfunction and breakdown [105,106,114]. Interestingly, it has been proposed that complimentary effects that AMPs elicit may be the cause of bacterial death, hence enhancing AMP efficiency and preventing the development of resistance [106,114]. Crucially, the contact between AMPs and the microbial membrane determines the antibacterial activity of AMPs independently of the precise mechanism of action and target location [110]. Artilysins are synthetic combinations of bacteriophage-encoded endolysins, which break down the peptidoglycans in the wall of bacterial cells, and certain amino acids (AMPs) that help the endolysin transduce through the outer membrane of Gram-negative pathogens to its target [115,116].

**Figure 3:**
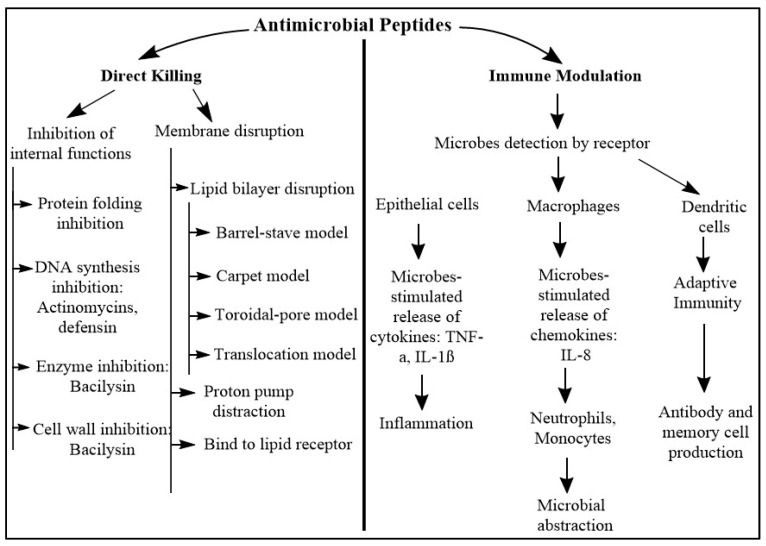
Mechanisms of action of AMPs. The left portion of the figure indicates the direct killing mechanism of AMPs, whereas the right side indicates the immune modulation mechanism of AMPs. In direct killing, AMPs either disrupt the pathogens’ cell membrane, or inhibit the internal functions like protein synthesis and cell wall formation, leading to cell death. Immune modulation is an indirect killing mechanism where the AMPs provoke the immune system to take actions against the target pathogen.

In addition to being strong antibiotics, AMPs have also been shown to be efficient inflammatory modulators and microbial toxin neutralizers [117]. AMPs exhibit a wide range of immunomodulatory actions, which collectively aid in the removal of pathogens from the host. These activities include chemotaxis stimulation, immune cell differentiation modification, and the start of adaptive immunity (Figure 3). In addition, the immunomodulatory actions reduce proinflammatory cytokine production mediated by toll-like receptors (TLRs) and/or cytokines and anti-endotoxin activity, which collectively prevent overwhelming and detrimental proinflammatory reactions, such as sepsis [24,110,118] (Figure 3). Different models have been given to describe the immune modulation mechanisms of AMPs. According to the “alternate ligand model”, AMPs directly bind to certain receptors on the cell surface, triggering receptor signaling [118]. According to the “membrane disruption model”, the AMPs locally change the region of the membrane that houses the receptor, which indirectly modifies its performance [110]. According to the “trans-activation model”, AMPs release a membrane-bound factor that may subsequently attach to its receptor [24]. Furthermore, it has been proposed that AMPs scavenge the endotoxin LPS, preventing LPS from binding to TLR4 and inducing inflammation [24].

## 5. Importance of Peptide Antibiotics

**Figure 4:**
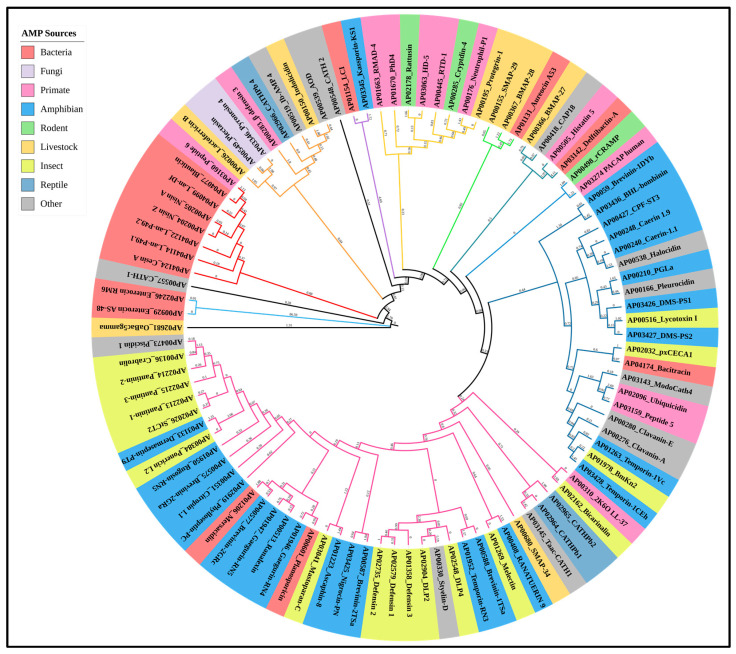
Phylogenetic analysis of 99 anti-MRSA antimicrobial peptides (AMPs) identified from different natural sources. Sources include different bacteria, fungi, animals, and plants. A total of 99 anti-MRSA AMP sequences were retrieved from the APD and Xuan et al. [125]. The phylogeny tree was constructed based on multiple sequence alignment using MEGA-11. The evolutionary history was inferred by using the Maximum Likelihood method and JTT matrix-based model. The tree with the highest log likelihood (−6284.65) is shown. Initial tree(s) for the heuristic search were obtained automatically by applying Neighbor-Join and BioNJ algorithms to a matrix of pairwise distances estimated using the JTT model, and then selecting the topology with superior log likelihood value. The tree is drawn to scale, with branch lengths measured in the number of substitutions per site.

The formation of AMPs in many species demonstrates their potential for antibacterial specificity, which can be applied in the goal of discovering new antimicrobials. AMPs have varying degrees of potency against different microbes; however, they also work in combination. The capacity of AMPs to interact with host cells and alter the immune response is another feature that may guide evolution [119], which may have implications for the health of the organism [120]. The efficiency of AMPs is determined by several factors, such as the target tissue, host microbiota, dosage, duration, administration method, formulation, and most medications. In particular, the regulation of AMP expression by gut microbiota lowers inflammation and maintains bacterial colonization [121]. By focusing on internal infections, autophagy regulation lowers the microbial burden and has anti-inflammatory properties [122]. AMPs can function as chemoattractants, which start the recruitment of innate immune cells to the infection site and trigger the production of chemokines by numerous type of immune cells, in a reciprocal connection between peptides and host immune factors [123]. In addition to identifying conserved and taxon-specific families, mapping the diversity and phylogenetic distribution would allow for the prediction of the evolutionary origins of some AMP families (Figure 4), for investigating substitutes for conventional antibiotics to restore the susceptibility of multidrug-resistant pathogens, and for making it easier to develop templates for the logical design of peptidomimetic drugs [122]. Additionally, the mapping may help identify several effective combinations to reduce antibiotic resistance. The injection of substances that induce the formation of natural peptides might potentially be one possible use [124]. Figure 4 shows the evolutionary relationship of anti-MRSA AMPs and the diversity of anti-MRSA AMPs within each group. AMPs have significant host functions in addition to their antibacterial properties. Additionally, they are quite important from a therapeutic standpoint.

### 5.1. Significance of Peptide Antibiotics in Microbial Host

AMPs possess a great deal of importance for their host, even though they manufacture all of these AMPs as a form of self-defense. AMPs have been linked to sporulation, germination, and several other cellular processes in microbes. Tyrothricin, an AMP, was found to suppress RNA synthesis and RNA polymerase in *B*. *brevis*, and it was proposed that this may be related to gene control during sporulation [126]. Using the peptide antibiotic gramicidin S, Frangou-Lazaridis et al. discovered a different result and concluded that gramicidin S inhibited transcription during germination and outgrowth but not during growth and sporulation [127]. Moreover, other AMPs were shown to cause sporulation in *B*. *brevis* cells maintained in culture conditions with low nitrogen sources [128]. Bacilysin, in contrast to other peptide antibiotics, also functions as a molecular messenger and influences a number of cellular processes, including spore quality [77,129]. By using NTG mutagenesis, a bacilysin-negative isolate of *Bacillus subtilis* PY79 was produced that was less dipicolinic acid-containing and more sensitive to heat, chemicals, and lysozymes than the initial wild-type isolate [130,131]. Bacilysin addition was used to increase the spore quality of these isolates up to the mid-log phase demonstrating how bacilysin affects *B*. *subtilis* PY79 spore quality [132]. Besides microbes, AMPs also play an important role in other hosts. Plant antimicrobial peptides (PAMPs) and insect antimicrobial peptides (IAPs) elicit defense against microbial attacks [133,134]. PAMPs exhibit distinct antimicrobial characteristics, such as fast killing, cell selectivity, and broad-spectrum activity, which render them attractive options for managing infections in humans and animals brought on by pathogens [134]. Similarly, IAPs have an antibacterial effect by breaking down the microbial membrane and prevent the development of drug resistance in microorganisms [133]. Moreover, AMPs identified in different natural sources are of great interest to researchers.

### 5.2. Clinical Importance

Antimicrobial peptides are promising molecules to treat multiple antibiotic-resistant microorganisms. They are effective against a broad range of microorganisms including bacteria, fungi, and viruses [37,135]. AMPs, for instance, tyrothricin, gramicidin S, vancomycin, and telavancin, are used as therapeutics in clinics [11,45,136]. The mode of action of these AMPs is such that they are supposed to target the membrane integrity, resulting cell burst and death. Current research demonstrates that this type of molecule employs additional methods to invade the target cells [12,137]. They can invade the target directly or by immune modulation [12,137]. In direct killing, they either target the cell membrane and kill the cell or they are internalized and inhibit different cellular processes, such as the synthesis of DNA and RNA [38,47]. In the case of indirect killing, AMPs activate the immune system, resulting in cell death [12,38]. Previous reports show that these AMPs are effective as antibiofilm, immune modulator, anticancer, and anti-inflammatory agents [27,62,113,135]. It has been observed that LL-37 and β-defensins, two well-known human AMPs of the cathelicidin and defensin families, respectively, attract immune cells and mast cells. [138], trigger monocytes, and provoke ion channel action in biomembranes, particularly in oocytes of *Xenopus laevis* [139]. Innate defense-regulator (IDR) peptide IDR-1, a promising AMP-active multidrug resistance Gram-positive and Gram-negative bacteria, did not attack the microorganisms directly. However, it acts through mitogen-activated protein kinase and other signaling pathways and improves monocyte chemokines while dropping proinflammatory cytokine responses [140]. IDR-1002, a synthetic cationic peptide, offers excellent defense against bacterial infections by immune modulation. It mainly acts through an increase in chemokine production and engagement of neutrophils and monocytes to the infection site [141]. Inflammation and the possibility of postoperative pathogen infection are common in cancer patients. Antibiotic resistance significantly affects the survival rate of cancer patients. AMP-based treatments are an appealing therapeutic alternative for cancer patients because of their direct anti-tumor efficacy and immunomodulatory properties. In clinical studies for the treatment of cancer, three AMPs have been investigated [142]. It is possible that traditional antibiotics can still completely kill the bacteria found in tumor cells. AMPs thus provide a novel approach to the therapy of cancer since they function as both antibacterial and anticancer peptides [143]. All of the previous studies indicate that AMPs are excellent alternatives to conventional antibiotics, making them a perfect candidate for further improvement. AMPs made synthetically or semi-synthetically or from organisms have been commercialized following successful trials, as described in Table 3. Table 4 shows the AMPs that are undergoing trials in different stages. Moreover, the AMP database shows that different AMPs that are effective against different microbes or cancer cells failed in clinical trials (http://dramp.cpu-bioinfor.org/, accessed on 24 February 2024).

## 6. Complications of Using Antimicrobial Peptides (AMPs) in Therapeutic Uses

A number of obstacles have been faced in the development of AMP-based treatment, such as stability, effectiveness, and toxicity, which restrict the clinical development of AMPs. In particular, the unfavorable pharmacodynamics of AMPs, which include their instability caused by the serum’s proteolytic enzymes breaking them down [143], the neutralization of their antitumor activity by negatively charged proteins and high/low density lipoproteins [144], and their rapid clearance by the kidney and liver [145], are relevant to their therapeutic uses. Numerous elements, including peptide length, net charge, hydrophobicity, and secondary structure, affect the action of AMPs. The length of peptides influences their antibacterial activity, and they must penetrate the lipid bilayer to keep the pore open [146]. However, the hydrophobicity and net-positive charge of the peptide also alter its length. The increased positive charge of AMP facilitates better peptide binding to anionic bacterial membranes [147]. Nevertheless, at high ionic strengths, the biological impact of highly charged peptides is greatly diminished [146].

Numerous AMPs have been studied in human clinical trials to prove their usefulness and safety, based on encouraging preclinical data. While some of these studies have been finished, stopped, or authorized, others are still in progress (http://dramp.cpu-bioinfor.org/). The stability, dosage optimization, and potential side effects of these AMPs depend on the length, sequence, and conformation of the tested AMPs [148]. As an example, a simple de novo synthesized peptide, pepD2, was used to evaluate the effects of remaining compounds in the newly synthesized peptide and modifications on the antibacterial activity against *A*. *baumannii*, stability in plasma, and toxicity to human HEK293 cells. With a Leu–Ile replacement, pepI2 can reduce the minimum bactericidal concentrations (MBCs) against *A. baumannii* by half (4 μg/mL). The half-life of a D-form peptide (pepdD2) in plasma was increased by more than 12 times, when the D-enantiomers of the Lys (K) and Leu (L) residues were substituted for the L-enantiomers. Compared to PepD2, PepD3 has a shorter 3-residue. Interestingly, peptide length reduction increased the IC50 to HEK293 cells; however, it had no effect on antibacterial efficacy [149]. A number of AMPs have cytotoxic and/or hemolytic effects at antimicrobial doses, which restricts their widespread application. One such AMP is polymyxins, which are essential antimicrobials for killing MDR Gram-negative bacteria but can be neurotoxic and nephrotoxic at high antimicrobial doses [150].

Some recent studies also reported the evaluation of AMP resistance in microbes [151,152]. Resistance mechanisms to AMPs have evolved for as long as AMPs have been around, making AMPs an old kind of protection. Both host–pathogen interactions and direct competition between bacterial species fuel the evolution of AMP resistance mechanisms. Due to their long history of producing AMPs, Gram-positive bacteria are thought to have evolved some of the first AMP resistance mechanisms [151]. Additionally, studies conducted on the model insect Tenebrio have shown that certain strains of AMP-resistant *S*. *aureus* performed better in the host [153]. In addition, over ten human-derived AMPs are being studied in clinical trials, which may cause cross-resistance to the host’s endogenous targets [154].

Combinations of AMPs were reported to be more active against microbes compared to the parental AMPs [155,156]. However, sometimes they are associated with higher hemolytic activity. The antibacterial potential of these combinations was diminished when modifications were made to lower the hemolytic activity [156]. A combination of cecropin A, LL-37, and magainin II showed excellent antibacterial activity against a broad range of bacteria including *S*. *aureus*, *E. coli*, *E*. *faecalis*, and *P*. *aeruginosa*; however, it showed higher hemolytic activity. The antibacterial activity of this combination was similarly decreased by its modification [156].

When it comes to clinical applications, their safety issues need to be tested. In vivo and in vitro tests show that only a few of these AMPs are non-hemolytic or hemolytic at minimal levels and safe for clinical applications [12,28]. Most of the AMPs are associated with toxicity and proteolytic degradation in clinical trials. Many of them were reported to be degraded rapidly by digestive enzymes and cleared quickly from the body after ingestion, reducing their half-lives [12,37,157]. Different policies have been studied to avoid these problems and to increase the effectiveness of AMPs, including structural modifications and vehicle delivery [158,159]. Many of these AMPs are still undergoing clinical trials for FDA approval [12]. OP145, an AMP similar to LL-37 of humans, has been undergoing trials and displayed higher antibacterial activity against bacterial ear infections [12]. APM pexiganan, which is derived from frogs, has been studied and found to be quite effective in treating diabetic foot ulcers [12]. However, the non-ribosomal AMPs were reported to be more effective in clinical trials compared to the AMPs identified from other hosts [157].

Emerging technologies, such as CRISPR and machine learning (ML) are amazing alternatives for AMP optimization and design. In order to utilize naturally occurring bacterial CRISPR–Cas systems for target-specific pathogen eradication, CRISPR–Cas technologies have been quickly applied to human cell gene editing, and animal model design for disease progression research [160]. In an effort to characterize antibiotic-resistant genes in clinical strains of *P. aeruginosa*, Chen et al. have refined a gene silencing technique based on Type I-F CRISPRi (CSYi). The study team was able to identify how the gene CzcR affects efflux pump genes, which are a key cause of *P*. *aeruginosa*’s multi-drug resistance [161]. The rapid generation and translation of AMPs may be facilitated by ML-based peptide modeling, which may also help to address some of the drawbacks associated with conventional drug discovery [162]. IK-16-1 is a new β-defensins-based AMP that has considerable antibacterial activity while preserving safety in host cells. IK-16-1 shows no hemolytic action and broad-spectrum antimicrobial capabilities against *C. albicans*, *P. aeruginosa*, *S. aureus*, and *E. coli* [8]. However, the application of CRISPR technologies to eradicate antibiotic resistance does not come without ethical issues and ramifications. One of the biggest obstacles to using CRISPR-based technologies in real time is the requirement for appropriate, approved criteria [160].

## 7. Designed Antimicrobial Peptides (AMPs) for Enhanced Efficacy

**Figure 5:**
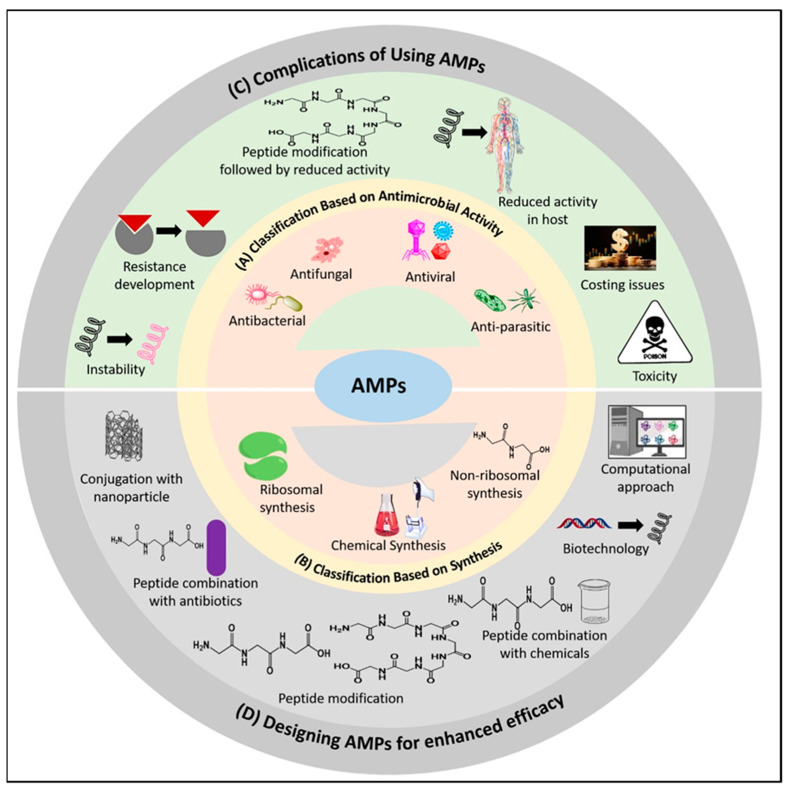
Schematic diagram of AMPs in combating superbugs. (**A**) The classification of AMPs based on antimicrobial activity. (**B**) The classification of AMPs based on synthesis. (**C**) Complications of using AMPs in clinical trials. (**D**) Designing AMPs for enhancing efficacy.

Researchers have implemented certain strategies to address the challenges posed by the use of AMPs. Some of them aim to forecast different AMPs’ structure or mechanism of action, while others try to increase their effectiveness or lower their toxicity. Cutting-edge technologies have made it possible to overcome complications in a relatively short time, and computational analysis has added another dimension to this area. The challenges of employing AMPs are described in Figure 5, along with potential solutions.

### 7.1. Structural Modification for Enhanced Performance

Peptide cyclization is a particularly promising method for enhancing AMPs’ stability and bioactivity [163]. When compared to monomeric KR-12 AMP, backbone-cyclized KR-12 dimers showed enhanced stability and antibacterial activity. The most potent cyclic dimer showed an 8-fold increase in fungicidal activity against *C. albicans* and a 16-fold increase in antibacterial activity against *P*. *aeruginosa* and *S. aureus* when compared to KR-12 [163]. By creating a β-sheet structure at the membrane surface, cyclic peptides attach to bacterial membranes with great force [164]. Compared to the original BSI-9, which had an MIC of 16–32 µg/mL, analogues of a cyclic AMP BSI-9 with a flexible linker showed better activity against *S*. *aureus* and *P*. *aeruginosa*, with MICs of 4 µg/mL and 8 µg/mL, respectively [165]. Utilizing the creation of disulfide bonds to establish an intramolecular cross-link between cysteine residues is another method of cyclization that improves protease stability [166]. A designed cyclic 17-amino acid-long β-defensin analogue that connects the internal hydrophobic domain of hBD1 and the C-terminal charged region of hBD3 by a disulfide bond showed excellent antibacterial activity against *P. aeruginosa*, *E. faecalis*, and *E. coli* and antiviral activity against herpes simplex virus type 1 [166]. One important method for imposing a peptide’s structure onto an α-helical by side chain linkage is stapling [167]. Magainin 2 (a class of antimicrobial peptide isolated from African frog *Xenopus laevis*) derivatives produced by stapling between the N-terminus’s first and fifth positions showed higher antimicrobial activity against both Gram-positive and Gram-negative bacteria than magainin 2, without exerting significant hemolytic activity [168].

### 7.2. AMPs with Lipid Nanomaterials

Because AMPs may self-assemble into organized amyloid-like nanostructures, their antibacterial activity is enhanced by building more powerful and focused contacts with microbial membranes [169]. The self-assembly of a histatin-based antimicrobial peptide and its self-assembling derivative were evaluated and they showed significant antibacterial activity against both Gram-negative and Gram-positive bacteria; however, no activity was found against fungi. Prokaryotes and eukaryotic microorganisms differ significantly in their antifungal and antibacterial peptide activity [169]. Nanomaterials effectively kill microbes by disrupting the bacterial cell membrane and resulting in intracellular substance leaking. In addition, nanomaterials can bind to a variety of bacterial cell constituents during membrane penetration, including DNA, ribosomes, and enzymes. The disruption of the regular physiological activities of cells causes oxidative stress, electrolyte imbalances, enzyme inhibition, and other bacteriostatic effects, which ultimately result in cell death [170]. Administering AMPs with the conjugation of nanoparticles (NPs) has gained more attention recently. Conjugated NPs offer AMPs a relatively wide surface area for adsorption and inhibit AMP self-aggregation [171]. Potential drug delivery nanocarriers have been found in nanostructures and possess two crucial characteristics in order for them to be effective drug carriers: they must be immunogenic and less cytotoxic. They do not require transfectants to be imported into the cytoplasm. Moreover, they may be transported by means of the endocytosis and exocytosis pathways without the assistance of multi-drug efflux pumps. Treatments based on nanotechnology can lessen the toxicity of AMPs to host tissue cells while increasing their stability and effectiveness. The tiny size, large surface area, and effective targeting capabilities of nanomaterials make them ideal for encapsulating AMPs [172,173].

### 7.3. Combination of AMPs

It was noted that the combined action of two AMPs was more potent against bacteria [155]. Given that biofilms are heterogeneous microbial communities, combination treatments seem especially appealing when targeting cells in various metabolic states (e.g., actively growing cells, dormant cells) and environmental conditions (e.g., acidic pH, lack of oxygen or nutrients) [155]. The “triple hybrid” of LL-37, melittin, and cecropin-A considerably increased the bactericidal action against a range of bacteria including *Bacillus anthracis*, *Burkholderia cepacia*, *Francisella tularensis* LVS, and *Yersinia pseudotuberculosis* [174]. By merging two distinct α-helical segments of BMAP-27 and OP-145, the Alzoubi group synthesized a novel hybrid peptide H4, which showed lowered toxicity profiles and a broad spectrum of action against both standard and multidrug-resistant bacterial strains in the range of 2.5–25 μM [156].

### 7.4. Combination of AMPs with Antibiotics or Chemicals

Combining antibiotics with AMPs can be a therapeutic strategy to combat antibiotic resistance, increase the effectiveness of medicines in killing off infections, and lessen the toxicity or side effects of antibiotics linked with concentration. This tactic can hinder the development of biofilms and bacterial survival by increasing the permeability of bacterial membranes, reducing the efflux of antibiotic drugs, and altering intracellular ion homeostasis [175]. When treating both plankton and biofilm cells, a number of AMPs, including HsAFP1, RsAFP2, and RsAFP1, showed synergistic action with the antimicrobial drugs [176]. Nisin, a polycyclic ampicillin derivative, when combined with penicillin, chloramphenicol, ciprofloxacin, indolicidin, or azithromycin, has demonstrated a synergistic effect on MRSA by inhibiting the formation of biofilms or preventing bacterial attachment to solid surfaces [177,178]. The most common combinations that produced synergistic interactions against MSRA isolates were nisin + daptomycin/ciprofloxacin, indolicidin + teicoplanin, and CAMA + ciprofloxacin [178]. Demeclocycline hydrochloride (DMCT) and the antimicrobial peptide SAAP-148 are tetracycline antibiotics that have been shown to exhibit synergistic antibacterial activity against the MDR *P*. *aeruginosa* strains PAO1 and ATCC27853. This suggests that DMCT and SAAP-148 combined therapy may be a useful strategy for treating MDR *P*. *aeruginosa*-related infections [179]. Plant-derived defensin HsAFP1, a phosphatidic acid-interacting peptide, can effectively reduce bacterial or fungal infection if it is coupled with the antibiotics caspofungin and amphotericin B [180]. Most remarkably, caspofungin and recombinant rHsAFP1 together exhibited synergistic efficacy against pre-grown *C*. *albicans* biofilms at doses as low as 0.53 μM. Up to 40 μM, rHsAFP1 was shown to be non-toxic to human HepG2 cells, confirming the prior finding that HsAFP1 lacked general cytotoxic action [180]. It is also possible to obtain the synergistic antimicrobial effects by mixing AMPs with other substances or medications. Nisin combined with citric acid had superior antibacterial activity against *S*. *aureus* and *L*. *monocytogenes* due to stronger morphological damage and increased release of cell constituents with fractional inhibitory concentration index values ranging from 0.25 to 0.375 and 0.19 to 0.375, respectively [181].

### 7.5. Genetic Modification

AMPs are promising next-generation antibiotics. To fully use their potential as antimicrobial agents, strategies for large-scale, reasonably priced manufacturing and purification will be needed. However, a significant barrier to AMP use continues to be the need for vast amounts of highly pure AMPs for fundamental research and clinical contexts. To get around this, genetic engineering techniques can be used to create enough different peptides in heterologous host systems. Recent studies showed several successful outcomes in producing AMPs in heterogenous organisms including bacteria, fungi, plants, and insects. The most often used microorganisms in microbial systems are yeast and bacteria because of their high cell densities, quick rates of development, and ease of manipulation [182]. Various AMPs, including adenoregulin, cecropin, crustin, defensin, hepcidin, histonin, human β defensin, lactoferrin, perinerin, thanatin, and viscotoxin, were expressed by bacterial species, including *E*. *coli*, *B*. *subtilis*, and *Propionibacterium freudenreichii* [183,184,185]. High levels of constitutive AMP expression resulted in harmful plant phenotypes, as demonstrated by tobacco plants that expressed fusion proteins encoding nine distinct AMPs. However, the detrimental effect was minimized by fusions to the cleavable carrier protein SUMO, as well as the cytotoxic effects of AMPs and fused AMPs [186].

### 7.6. Computational Approach

AMPs can be created using a variety of computer techniques, including genetic algorithms, machine learning techniques, and software analysis. These methods incorporate vital data regarding the biochemical characteristics and bioactivities of AMP sequences. As a result, before a sequence is synthesized, its antibacterial potential can be predicted. The development of different web-based software has made it easier to analyze and predict AMPs using a huge array of databases [187,188,189]. A machine learning algorithm was integrated into a web server known as “ABP-Finder”, which is among the most advanced AMP predictors available today. It has demonstrated exceptional precision in identifying potential peptide hits against *P*. *aeruginosa* when scanning sizable databases like the human urine peptidome [189]. As a first step towards the de novo design/optimization of antibacterial peptides (ABPs), ROSE (Random Model of Sequence Evolution) generates diversity-oriented libraries of peptides by blocking *E*. *coli* FoF1-ATP synthase [188]. For instance, the lead peptide LI14 and a panel of synthetic peptide compounds with unique structures based on database filtering technology (DFT) have strong antibacterial efficacy against drug-resistant *E*. *coli* and MRSA. Rapid bactericidal action, good anti-biofilm and -persisters activity, and a low potential to generate resistance are all displayed by LI14. Furthermore, LI14 exhibits resistance to changes in pH, temperature, and pepsin treatment, and it exhibits no observable toxicity in vivo or in vitro [190]. RNA sequencing (RNA-seq.) datasets may be used to detect AMP sequences using rAMPage, a scalable bioinformatics tool. RNA-seq. datasets from amphibian and insect species that are publically available have been used to thoroughly assess rAMPage. Of the 1137 putative AMPs found, 1024 were deemed new based on homology criteria. Seven of the twenty-one peptides that were examined for antibacterial susceptibility against S. aureus and *E*. *coli* showed strong activity [191]. Additionally, in order to encounter multidrug-resistant fungal diseases that threaten immunocompromised individuals, it is imperative to create antifungal medicines that target intracellular molecules. Through computational and experimental investigations, an anticryptococcal AMP was recently created and tested, and more information on its mode of action against Cryptococcus neoformans was presented [192].

## 8. Conclusions

Antimicrobial peptides (AMPs) represent a promising frontier in the battle against the escalating crisis of antibiotic resistance, as “peptide antibiotics” [193]. With their broad-spectrum antimicrobial properties and unique mechanisms of action, AMPs offer a viable alternative to traditional antibiotics, which are rapidly losing their effectiveness due to the emergence of multidrug-resistant pathogens. The development of AMPs as therapeutic agents is accompanied by several challenges, including stability, specificity, toxicity, and high costs of production. However, advancements in computational biology, structural modifications, and novel delivery systems are paving the way for overcoming these obstacles. The design and optimization of synthetic AMPs, using strategies such as peptide cyclization, stapling, and nanomaterial conjugation, have shown significant improvements in enhancing stability and antimicrobial efficacy while minimizing adverse effects. Despite the promising potential of AMPs, their clinical translation requires rigorous evaluation to ensure safety and efficacy. The development of AMPs must consider the intricate balance between their antimicrobial activity and potential cytotoxicity to human cells. Future research should focus on large-scale production, comprehensive clinical trials, and the development of delivery systems that enhance the bioavailability and stability of AMPs in physiological conditions. Finding a more effective biological processing technique, cutting costs, and raising yields are significant issues in real-world applications. Furthermore, in order to facilitate future mass manufacturing, it will be necessary to investigate the organism’s own AMP expression and create a better expression vector when new AMPs in nature are discovered. Further research into the reported AMPs is necessary to overcome the structure–function interaction issue. Emerging technologies in particular, CRISPR and machine learning, may add additional advantages in the optimization and design of AMPs. Undruggable proteins are frequently distinguished by large, intricate structures or activities that are challenging to disrupt using traditional drug design techniques. Targeting such unreachable locations has drawn significant medical attention and is thought to be a significant direction for treating human illnesses. Further efforts using ML or AI could help in designing and optimizing AMPs to target these undruggable proteins. Given the limited success rate of the clinical use of AMPs at the moment, medical research in the near future must be accompanied by promising innovations.

## Figures and Tables

**Table 1:** List of antimicrobial peptides naturally found in plants, animals, and insects.

Class	Peptide	Amino Acid Sequence ^a^ and Length	Producer	Active Against	Mode of Action	Ref.
Cecropin	Cecropin A	KWKLFKKIEKVGQNIRDGIIKAGPAVAVVGQATQIAK (37)	*Ascaris suum*	Gram-negative bacteria	Interactions with nucleic acids	[25]
	Cecropin B	KWKVFKKIEKMGRNIRNGIVKAGPAIAVLGEAKAL (35)	*Hyalophora cecropia*	Multidrug-resistant cancer cells, and bacteria	Form pores in CM	[26,27]
	Cecropin D	^b^ MNFTKILFFVVACVFAMRTVSAAPWNPFKELEKVGQRVRDAVISAGPAVATVAQATALAKGK (62)	*H. cecropia*	Gram-negative bacteria	Disruption of CM	[28]
	Papiliocin	RWKIFKKIEKVGRNVRDGIIKAGPAVAVVGQAATVVK-NH2 (37)	*Papilio xuthus*	Gram-negative bacteria, anti-inflammatory	Disruption of CM	[29,30]
	Cecropin P1	SWLSKTAKKLENSAKKRISEGIAIAIQGGPR (31)	*Ascaris suum*	Bacteria, multidrug resistant cancer cells	Disruption of CM	[27]
Cathelicidin	hCAP-18	EFKRIVQRIKDFLRNLV (17)	*Homo sapiens*	Cell growth, migration, and antimicrobial	Disintegration of CM molecules	[31,32]
	Cathelicidin-AL	RRSRRGRGGGRRGGSGGRGGRGGGGRSGAGSSIAGVGSRGGGGGRHYA (48)	*Amolops loloensis*			[33]
	Fowlicidins 1,2,3 and cathelicidin Beta-1	RVKRVWPLVIRTVIAGYNLYRAIKKK (26) (Fowlicidin-1, chain A)	*Gallus gallus domesticus*			[31]
	Protegrin 1	RGGRLCYCRRRFCVCVGRX (19)	*Sus* sp.			[34]
Defensin	cis-defensins (e.g., plant defensin 1)	RECKTESNTFPGICITKPPCRKACISEKFTDGHCSKILRRCLCTKPC (47)	*Nicotiana* sp.	Bacteria, fungi, enveloped and nonenveloped viruses	Damages the CM	[35]
	trans-defensins (e.g., human β-defensin)	KKCWNGGRCRKKCKENEKPIGYCRNGKKCCVN (32)	*H*. *sapiens*		Disruption of CM, inhibition of DNA and RNA synthesis	[36,37,38]
Magainins	Magainin-2	GIGKFLHSAKKFGKAFVGEIMNS (23)	*Xenopus laevis*	Bacteria, protozoa, fungi, virus, and cancer	Disrupts the CM	[37,39]

^a^ One-letter amino acid code with the following additions. Sequences of the structures were collected from the protein database (PDB) (https://www.rcsb.org/, accessed on 15 January 2024). ^b^ Sequence was collected from UniProt (https://www.uniprot.org/, accessed on 15 January 2024).

**Table 2:** List of antimicrobial peptides naturally synthesized by microorganisms.

Domain	Peptide	Producer	Activity Against	Mode of Action	Ref.
Bacteria	Nisin	*Lactococcus lactis*	Gram-positive bacteria	Perturbation of the PM	[40,41]
	Vancomycin	*Amycolatopsis orientalis*	Gram-positive bacteria	Inhibition of CW synthesis	[41]
	Telavancin	Derivative of vancomycin	Gram-positive bacteria	Inhibition of CW synthesis	[42]
	Gramicidin S	*Bacillus brevis*	Bacteria and fungi	Membrane deformation	[43]
	Bacitracin	*B. subtilis*	Gram-positive bacteria	Interfere peptidoglycan biosynthesis	[44]
	Tyrocidine	*B. brevis*	Gram-positive bacteria	DNA damage, and interfere with DNA-binding proteins	[45]
	Polymyxin B	*B. polymyxa*	Bacteria	Alters outer membrane permeability	[46]
	Bacillomycin, Fengycins, Surfactines, Bacillibactin, and Difficidin	*Bacillus* sp.	Bacteria and fungi	Damage CW and CM, binds to genomic DNA leading to cell death	[47]
	Bacilysin	*Bacillus* sp.	Bacteria, fungi, algae, and protozoa	Inhibits glucosamine 6-phospate synthase	[48]
	Iturins	*B*. *subtilis*	Fungi	Leakage of K^+^ and other vital ions	[49]
	Syringomycin	*Pseudomonas syringae*	Bacteria, fungi, and virus	K^+^ and H^+^ influx leads to cell death	[50,51]
	Ecomycins	*Pseudomonas viridiflava*	Fungi	Unknown	[51]
	Pseudomycins	*P. syringae*	Fungi	Unknown	[51]
	Daptomycin	*Streptomyces roseosporus*	Gram-positive bacteria	Disrupts bacterial cell membrane	[52]
	Albonoursin	*Streptomyces noursei*	Bacteria	Unknown	[53]
	Amphomycin	*Streptomyces canus*	Gram-positive bacteria	Inhibit peptidoglycan biosynthesis	[54]
	Munumbicins	*Streptomyces* NRRL 30562	Bacteria, fungi, parasite, and cancer cells	Unknown	[51]
	Kakadumycins	*Streptomyces* NRRL30566	Bacteria, fungi, parasite, and cancer cells	Inhibit RNA synthesis.	[51,55]
	Xiamycins	*Streptomyces* GT2002/1503	Bacteria and fungi	Unknown	[51,56]
Fungi	Copsin	*Coprinopsis cinereacopsin*	Gram-positive bacteria	Inhibitor of CW synthesis	[57]
	Peptaibol	*Trichoderma*	Bacteria and fungi	Membrane leakage	[58]
	Plectasin	*Pseudoplectania nigrella*	Bacteria	Acts by binding to bacterial cell wall precursor Lipid II	[59]
	Echinocandins	*Aspergillus rugulovalvus*, *Zalerion arboricola*, *Papularia sphaerosperma*	Fungi	Inhibit glucan synthase	[60]
	Aculeacins	*Aspergillus aculeatus*	Fungi	Inhibit CW biosynthesis	[61]
	Aureobasidin	*Aureobasidium pullulans*	Fungi	Inhibition of actin and chitin assembly and synthesis of sphingolipids	[61]
	Yeast killer toxins	*Saccharomyces cerevisiae*	Fungi	Form cation channels in cell membrane, enter into nucleus, and interact with other proteins	[49]
Archaea	VLL-28	*Sulfolobus islandicus*	Bacteria and fungi	Membrane damages and nucleic acid binding	[62]
	PaDBS1R6	*Pyrobaculum aerophilum*	Bacteria and cancer cell	Unknown	[63]

Note: CW: cell wall; PM: plasma membrane; and CM: cell membrane.

**Table 3:** List of commercially available AMPs on the market (http://dramp.cpu-bioinfor.org/).

Name	Description	Active Against	Company
Daptomycin	Synthetic cyclic lipopeptide antibiotic	*S. aureus* infections (bacteremia)	Cubist Pharmaceuticals LLC (Lexington, MA, USA) (Merck and Co., Rahway, NJ, USA)
Dalbavancin	Semisynthetic lipoglycopeptide and antibacterial	Acute bacterial skin infections, osteomyelitis and septic arthritis	Allergan (Dublin, Ireland) (formerly Actavis and Durata Therapeutics)
Telavancin (TD-6424)	Semi-synthetic derivative of vanocymycin	MRSA and other Gram-positive bacteria	Clinigen Group plc (Burton-on-Trent, UK)/Innoviva Inc. (South San Francisco, CA, USA)/Pendopharm (Montreal, Canada)/Theravance Biopharma Inc. (South San Francisco, CA, USA)/University of Illinois
Oritavancin	Glycopeptide antibiotic used to treat Gram-positive bacteria	Acute bacterial skin infection	The Medicines Company (Parsippany-Troy Hills Township, NJ, USA)
Bacitracin	Bacitracin A is an antibacterial homodetic cyclic peptide	Wound infections, pneumonia, empyema in infants, skin and eye infections	Unknown
Colistin (polymyxin-E)	To treat Gram-negative bacteria	Gram-negative bacilli, particularly *P*. *aeruginosa*	Unknown
Polymyxin B	Basic polypeptides of about eight amino acids that display cationic detergent action on cell membranes	*P*. *aeruginosa* infections	Unknown
Tyrothricin	A polypeptide antibiotic mixture obtained from *Bacillus brevis*	Skin and oropharyngeal mucous membranes infections	Unknown
Vancomycin	A glycopeptide antibiotic	*S. aureus* infections	Unknown
Gramicidin S	Cyclic peptide biosynthesized from *Bacillus brevis*	Potent against Gram-negative and Gram-positive bacteria and fungi	Unknown
Gramicidin D	A heterogeneous mixture of three antibiotic compounds, gramicidins A, B, and C	Skin and eye infections	Unknown

**Table 4:** List of AMP in clinical trials (http://dramp.cpu-bioinfor.org/).

Name	Description	Active Against	Stage of Development
Histatin	Antifungal	Oral candidiasis	Phase II-III
PAC113	Antifungal	Oral candidiasis in HIV patients	Phase IIb
Plectasin	Fungal defensin	Pneumococcal and streptococcal infections	Phase I
D2A21	A 22-residue α helix peptide	Skin infections against multidrug-resistant pathogens	Phase III
Glutoxim (NOV-002)	Antibacterial	Tuberculosis	Phase III
PMX-30063	Defensin structural mimetic	*Staphylococcus* spp.	Phase II
Surotomycin	Antibacterial agents for the treatment of Gram-positive infections	Diarrhea and *Clostridium Difficile* infections	Phase III
PL-5	Has very strong bactericidal activity	*P*. *aeruginosa*, and MRSA and multi-drug resistant *A*. *baumannii* containing NDM-1 gene	Phase IIIb
Murepavadin	Synthetic by amino acid substitution of protegrin I	Nosocomial and ventilator-associated bacterial pneumonia	Phase III
Sifuvirtide	Antiviral designed peptide	HIV fusion inhibitor	Phase II
Enfuvirtide and Fuzeon	Enfuvirtide is a 36 amino acid biomimetic peptide that is structurally similar to the HIV proteins	HIV infection	Approved by FDA (Trimeris)
Cefilavancin	Covalently linked glycopeptide–cephalosporin (β-lactam) heterodimer antibiotic and active against Gram-positive bacteria	Gram-positive infections, skin and soft tissue infections	Phase III
Lotilibcin	Lipodepsipeptide	MRSA	Phase I/II

## Data Availability

Not applicable.
